# Morphological and Molecular Characterization of a Fungus, *Hirsutella* sp., Isolated from Planthoppers and Psocids in Argentina

**DOI:** 10.1673/031.013.1801

**Published:** 2013-03-15

**Authors:** Andrea V. Toledo, María E. Simurro, Pedro A. Balatti

**Affiliations:** 1 Centro de Investigaciones de Fitopatología (CIDEFI). Facultad de Ciencias Agrarias y Forestales. UNLP. Calle 60 y 1 19 s/n, 1900, La Plata, Buenos Aires, Argentina

**Keywords:** Cixiidae, Delphacodes kuscheli, Ectopsocidae, entomopathogenic fungi, Hirsutella citriformis, internal transcribed spacer

## Abstract

A mycosed planthopper, *Oliarus dimidiatus* Berg (Hemiptera: Cixiidae), and two psocids, *Heterocaecilius* sp. (Psocodea: Pseudocaeciliidae) and *Ectopsocus* sp. (Ectopsocidae), were collected from Los Hornos and La Plata, Buenos Aires, Argentina between February and September 2007. Observations of mycelia growing on the host revealed that the putative fungal parasite had synnemata supporting monophialidic conidiogenous cells. Likewise, *in vitro* fungal cultures presented characteristics typical of the fungus *Hirsutella citriformis* Speare (Ascomycota: Hypocreales: Clavicipitaceae). The identity of the isolated fungi characterized based on morphological aspects was complemented by means of the internal transcribed spacer sequences. The sequences of both isolates were highly homologous to those of *Cordyceps* sp. (Fries) Link and *Ophiocordyceps sinensis* (Berkely) G.H. Sung, J.M. Sung, Hywel-Jones, and Spatafora (Ophiocordycipitaceae). We additionally confirmed that both isolates had the ability to infect and kill adults of *Delphacodes kuscheli* Fennah (Hemiptera: Delphacidae) after 10 days. Therefore, based on the morphology of the isolated fungi, their ribosomal internal transcribed spacer sequence, and their ability to parasite insects, we conclude that the fungi isolated belong to the genus *Hirsutella* and might have biotechnological potential.

## Introduction

A great number of insects are always threatening crops of economic importance such as maize, wheat, soybean, etc. They may affect plants either directly by causing mechanical damags throughout feeding, or indirectly by transmitting and spreading plant pathogens such as viruses, mollicutes, and other bacteria. However, pesticides are not the best alternative of control, among other things, because of their undesirable effect upon the environment, altering ecological features. In addition, insects usually develop resistance to chemical pesticides, leading in some cases to the use of chemical pesticides more frequently and at higher concentrations, which might worsen the problem (Watkins et.al 2012). Based on the negative effects of pesticides and the fact that sustainable agriculture requires better strategies to maintain insect populations under threshold values, biological control appears to be a potential tool for controlling insects. Entomopathogenic fungi might be a useful tool to develop an Integrated Pest Management program.

The use of entomopathogenic fungi as a microbial control agent was considered for the first time at the end of the 19th century, and the idea was widely accepted among researchers at a time when synthetic chemical insecticides were still unknown. However, the highest number of studies was carried out during the 20th century (Cantwell 1974; Burges 1981; Davidson 1981; Ferron 1985). Since then, fungi have been found to have certain advantages over other methods of controls because they have restricted host range and are harmless to non-target organisms such as predators, parasites, and other pathogens (Goettel et al. 1990, 2001; Lacey and Goettel 1995; Goettel and Hajek 2001; Vestergaard et al. 2003). Therefore, biocontrol with fungi is advantageous and might be used within Integrated Pest Management programs. Furthermore, they can be combined with several fungicides and many other types of pesticides (Wraight et al. 2007).

Entomopathogenic fungi are natural enemies of arthropods worldwide, occupying virtually every niche in which arthropods are found, showing their potential to be used to formulate products for biological control (Hajek and St. Leger 1994; Shah and Pell 2003). There are approximately 750 species of entomopathogens within the fungal kingdom; most of them belong to the Zygomycota and Ascomycota divisions (Hajek 1997). Most entomopathogens within Zygomycota occur within the order Entomophthorales, that is characterized by the formation of sexual spores (zygospores). While most species are obligate parasites with a restricted host range, some species, such as *Zoophthora radicans* (Brefeld) Batko, have relatively wide ones (Wraight et al. 2007). The most common genera include *Conidiobolus, Entomophaga, Entomophthora, Erynia, Pandora, Neozygites*, and *Zoophthora*. A few genera of entomopathogenic fungi produce an ascomycetous sexual state (i.e., ascospores produced within an ascus), characteristic of the Ascomycota division. The most important of these genera include *Cordyceps, Torrubiella*, and *Ascosphaera*. Many species of entomopathogenic fungi appear to have lost most or all of their capacity to produce a sexual state. These fungi include prominent pathogens in the genera *Aspergillus, Aschersonia, Beauveria, Culicomyces, Fusarium, Gibellula, Hirsutella, Hymenostilbe, Lecanicillium, Metarhizium, Nomuraea, Isaria, Sorosporella*, and *Tolypocladium*. All of the asexual (anamorphic) forms of these fungi produce spores, termed conidia, and some produce chlamydospores (Wraight et al. 2007).

*Hirsutella* is one of the most abundant and important entomogenous fungi (Liang et al. 2005) and might play an important role in the control of pest insects in nature (Evans 1974, 1982). The genus *Hirsutella* was erected by Patouillard (1892), based on the type species *H. entomophila* Pat., which he described from a specimen collected on a beetle in Ecuador. Minier and Brady (1980) divided this entomogenous genus in two sections (Synnematous and Mononematous) based on the presence or absence of synnemata. Most species of *Hirsutella* are synnematous, a few are mononematous, and some others occasionally produce synnemata. Many members are believed to be anamorphs of teleomorphs within the genera *Cordyceps* and *Torrubiella* (Petch 1935; Mains 1951; Kendrick and Carmichael 1973; Hywel-Jones 1995a, b, 1997; ). They include more than 90 species that infect and parasitize a variety of invertebrate species, such as mites, nematodes, and insects, many of which are economically important pests (Seifert and Boulay 2004). Although *H. thompsonii* F.E. Fisher, a parasite of citrus rust mite, was used to make commercial formulations in the USA, the use of representatives of this genus to control pests is limited because many of these pathogens are fastidious organisms that require specific conditions to grow *in vitro* (Boucias et al. 2007a).

In Argentina, there is increasing concern regarding the use of chemicals to control insects, therefore many labs and companies are developing natural products to control pests. Regarding this development, it is important to have a wide array of characterized entomopathogenic fungi. However, little is known about the presence and fungal-host in- teraction of species of *Hirsutella* in Argentina. There is only one report by Toledo et al. (2008) describing the presence of *H. citriformis* Speare (Ascomycota: Hypocreales: Clavicipitaceae) and *H. strigosa* Petch infecting *Doru lineare* (Eschscholtz) (Dermaptera: Forficulidae) and *Ectopsocus californicus* (Banks) (Psocodea: Ectopsocidae), respectively. As part of a program aimed at developing biocontrol tools, entomopathogenic fungi are being isolated from different insects. Here, we report the isolation of representatives of the genus *Hirsutella* on naturally infected insects belonging to the orders Hemiptera and Psocodea. We identified and characterized them by means of morphological and molecular characters. Additionally we confirmed that both *Hirsutella* strains infected and parasitized *Delphacodes kuscheli* Fennah (Hemiptera: Delphacidae), the major vector of *Mal de Río Cuarto* virus, a severe endemic disease in corn crops in Argentina.

## Materials and Methods

### Isolation and preservation of fungal pathogens

Between February and September 2007, mycosed adults of *Oliarus dimidiatus* Berg (Hemiptera: Cixiidae) and *Heterocaecilius* sp. (Psocodea: Pseudocaeciliidae), and a nymphal *Ectopsocus* sp. (Psocodea: Ectopsocidae) were collected from rice, *Oryza sativa* L. (Poaceae), from Los Hornos, Buenos Aires, Argentina (34° 52′ S, 57° 58′ W), and from *Ligustrum lucidum* Ait. (Oleaceae) from La Plata, Buenos Aires, Argentina (34° 54′ S, 57° 56′ W), respectively. Infected insects were placed in sterile plastic containers and were taken to the laboratory to isolate fungi. These insects were so badly damaged that some essential elements for their identification at the species level were lacking. Fungal cultures were obtained from monosporic isolates in the manner described by Lecuona (1996) and incubated on Sabouraud dextrose agar +1% yeast extract (SDAY 1%) at 26° C in the darkness for 15 days. Mycosed planthoppers were deposited as herbarium material in the Mycological Collection of the Institute of Botany Carlos Spegazzini (LPSC, La Plata, Buenos Aires, Argentina) under the accession number LPSC 47786. Subcultures of the fungi, isolated from planthoppers and psocids, were deposited in the USDA-ARS Collection of Entomopathogenic Fungal Cultures under the accession numbers ARSEF 8378 for planthoppers, and ARSEF 8677, 8678, and 8679 for psocids.

### Identification and morphological characterization of fungal pathogens

Fungal structures such us conidia and conidiogenous cells obtained from dead insects were measured to enable specific identification. Mycelia were mounted in lactophenol cotton blue (0.01% w/v) and observed with a Wild M20 microscope. Infected insects were photographed using a Wild M5 stereo microscope fitted with a Sony Cyber-shot DSCW100 digital camera (www.sony.com). Fungal preparations were photographed using a Nikon YS2-H microscope (www.nikon.com) equipped with a Nikon D40 digital camera. Monosporic isolates cultured on SDAY 1% were incubated at 26° C in darkness for 21 days. Growth rates and aspects of the colony were recorded. Growth was estimated based on radial growth of the colony, which was measured by drawing two orthogonal diameters at 7, 14, and 21 days after incubation.

### DNA sequencing

The identification of the isolated fungi based on morphological characteristics was complemented by means of the internal transcribed spacer (ITS) sequences. This DNA fragment included the 3′ end of the 18S rDNA, ITS1, the 5.8 rDNA, ITS2, and the 5′ end of the 28S rDNA. Genomic DNA was isolated from monosporic fungal cultures by conventional procedures (Sambrook and Russell 2001). The ITS was amplified using primers ITSl and ITS4 (White et al. 1990). Amplification reactions were performed in a 50-µl reaction volume under the following PCR cycling conditions: one cycle of denaturation at 95° C for 3 min, followed by 34 cycles of denaturation at 95° C for 1 min, annealing at 52° C for 30 sec, and elongation at 72° C for 1 min, with a final extension step of 72° C for 10 min. The PCR products of approximately 550 bp length were resolved in 1% agarose gels stained with ethidium bromide. The PCR products were sequenced by mean of the mentioned primers in an Applied Biosystem 3130 sequencer (www.appliedbiosystems.com) based on the procedure described by Sanger et al. (1977).

### Phylogenetic analysis

The ITS sequence of both *Hirsutella* strains were compared with those of 15 species of entomopathogenic *Hirsutella* and its related genera available in the National Center for Biotechnology Information (NCBI) gene bank (Figure 2). Sequences were aligned using the clustal W BioEdit version five. Maximum parsimony analysis was performed and the branches were supported by the bootstrap method (100 replicates). A dendrogram was generated based on the rDNA sequences by means of the Mega5 Software (www.megasoftware.net) (Tamura et al. 2011).

### Infectivity tests

The ability of *Hirsutella* strains to infect *D. kuscheli* was evaluated on live insect specimens that had been collected from oat, *Avena sativa* L. (Poales: Poaceae), grown in plastic flowerpots and isolated inside 24 cm × 9 cm polyethylene terephthalate plastic cages in a greenhouse. Adults of *D. kuscheli* were placed inside glass vials and then introduced onto Petri dishes containing fungal cultures. In order to inoculate specimens of the insects with conidia, they were placed for 5 min over the synnemata. A total of 20 insects were inoculated with each fungal isolate, and 20 noninoculated insects were used as controls. Both fungal isolates were grown on SDAY 1% for 60 days al 26° C in darkness. After inoculation, insects were incubated at 26° C, at a high relative humidity (> 90%), and with a photoperiod of 14:10 L:D, inside polyethylene terephthalate plastic bottles containing young oat plants according to Toledo et al. (2007). Young oat plants were changed every three days. Insects were checked every 24 hr up to 14 days. Dead insects were removed daily and superficially sterilized placing the specimens in 70% ethanol for a few seconds, washed in sterile distilled water, placed in 0.5% sodium hypochlorite, and finally rinsed again in distilled water according to Lacey and Brooks (1997). Then, they were placed in Petri dishes containing moistened filter paper with sterile distilled water and incubated at 26° C for 3–5 days. Mycelia emergence was confirmed microscopically.

## Results

### *Hirsutella* sp. isolate examined: ARSEF 8378

**Figure 1.**
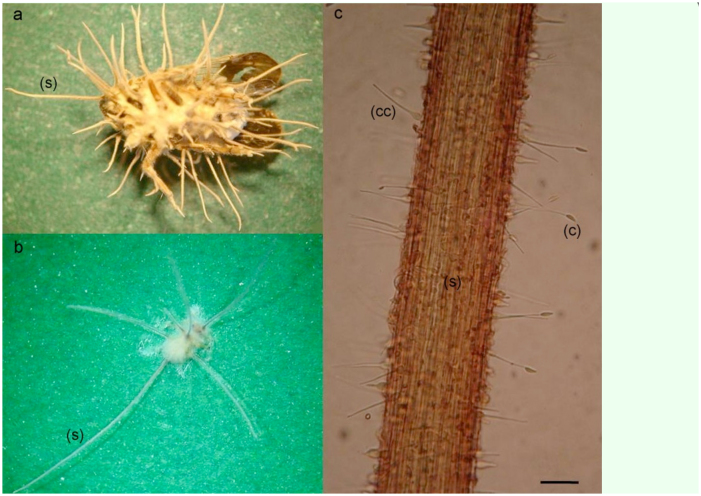
a: Adult *Oliarus dimidiatus* infected with *Hirsutella* sp. ARSEF8378. Note the formation of synnemata (s) branched with short lateral branches, b: Nymph of *Ectopsocus* sp. infected with *Hirsutella* sp. ARSEF8679. Note the synnemata (s) is slender and simple, c: Conidiogenous cells (ce) and (c) conidia of *Hirsutella* sp. ARSEF8679 arising as lateral cells over the whole length of the synnema (s). Scale bar: a: 2.5mm, b: 2mm, c: 17µm. High quality figures are available online.

Fungal infected insects were fully covered with a brownish mycelium that kept them adhered to the substrata. Fungal cultures presented characteristics typical of fungi belonging to the genera *Hirsutella*. Synnemata were slender, simple, or branched with short lateral branches (Figure 1a), composed of a compact bundle of longitudinal hyphae, and pubescent from the conidiogenous cells. Conidiogenous cells formed a moderately compact layer over the surface of the synnema, mostly arising as lateral cells produced over the whole length of the synnema, and were intercalary along the length of the mycelial strands (Figure 1c). Furthermore, they were monophialidic, with the ellipsoid base tapering abruptly to long phialidic sterigmata, 35.6–55.4 µm long (44 ± 1.3 µm; *n* = 20) and 3.9–4.9 µm wide at the base (4.6 ± 0.09 µm; *n* = 20) (Figure 1c). Sterigmata were about 28.7– 47.5 µm long (36.7 ± 1.3 µm; *n* = 20). Conidia were hyaline, aseptate, smooth-walled, fusiform or elliptical, solitary or occasionally paired, and 5.9–7.9µm (7 ± 0.09 µm) × 2–3 µm (2.5 ± 0.07 µm; *n* = 30) (Figure 1c). Colony diameters, after 7, 14, and 21 days of incubation, were 1.2–1.6 cm (1.5 ± 0.04 cm), 3.1–3.7 cm (3.5 ± 0.06 cm), and 4.6–4.9 cm (4.8 ± 0.04 cm; n = 11), respectively. When cultured *in vitro*, cultures had a white to brownish appearance, with the presence of yellowish to brownish exudates drops, and did not form synnemata after three weeks of incubation.

Specimens examined: Adult *O. dimidiatus* collected from *O. sativa*, Los Hornos, Buenos Aires, Argentina, 14 February 2007. Herbarium material access number LPSC 47786.

### *Hirsutella* sp. isolate examined: ARSEF 8679

A brownish mycelium fully covered the insect, attaching it to the substratum. It produced slender and simple synnemata (Figure 1b), composed of a compact bundle of longitudinal hyphae, which were pubescent from the conidiogenous cells that mostly arose as lateral cells over the whole length of the synnema. Conidiogenous cells were monophialidic with an ellipsoid base, tapering abruptly to long phialidic sterigmata, which were 22.4– 34.7 µm long (31 ± 0.6 µm; n = 30) and 3.4– 4.5 µm wide at the base (4.1 ± 0.07 µm; n = 30). Sterigmata length was 16.8–28 µm (24.2 ± 0.4 µm; n = 30). Conidia were hyaline, aseptate, smooth-walled, fusiform or elliptical, solitary or occasionally paired, and 5.6–7.8 µm (6.8 ± 0.1 µm × 2.2–2.8 µm (2.3 ± 0.03 µm; n = 30). Colony diameters, after 7, 14, and 21 days of incubation, were 1.4–1.6 cm (1.5 ± 0.03 cm), 3.3–3.6 cm (3.4 ± 0.05 cm), and 4.5–4.7 cm (4.6 ± 0.03 cm; n = 11), respectively. Cultures appeared white to brownish and did not form synnemata after three weeks of incubation.

Specimens examined: Nymphal *Ectopsocus* sp. collected from the underside of living leaves of a *L. lucidum* tree, La Plata, Buenos Aires, Argentina, 24 September 2007.

### DNA sequencing and molecular analysis

**Figure 2.**
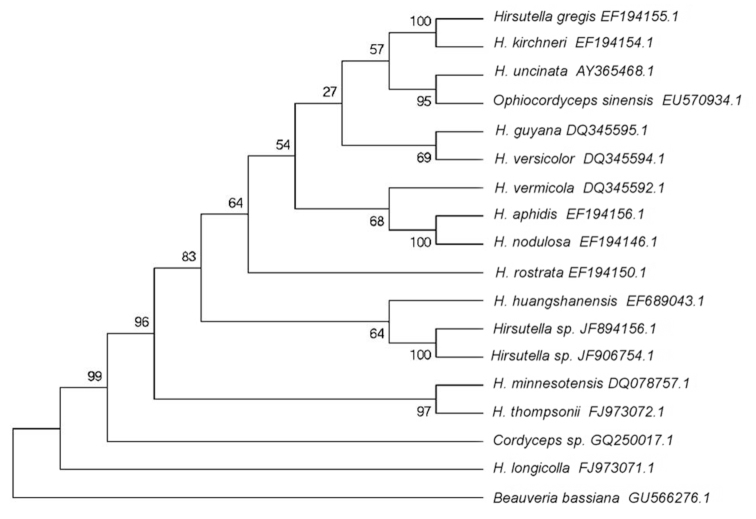
Maximum parsimony tree of the internal transcribed spacer nucleotide sequences generated from a common group of *Hirsutella* species and its related genera. Species names are followed by the GenBank accession numbers. High quality figures are available online,

The ITS is a conserved rDNA sequence that has been widely used both alone and in combination with other universal sequences, such as tubulin, actin, etc., to identify, characterize, and perform phylogenetic analysis of fungal isolates (Bałazy et al. 2008). The sizes of the ITS sequences determined for both *Hirsutella* sp. ARSEF 8378 and ARSEF 8679 were deposited in the GenBank under the accession numbers JF894156 and JF906754 respectively, were 510 bp, and differed from each other by only two substitutions. By means of the NCBI BLAST algorithm, we found that both ITS sequences were highly homologous to those from species in the phylum Ascomycota, class Sordariomycetes (Figure 2). Furthermore, the most significant alignment was the sequence from *Cordyceps* sp. and *Ophiocordyceps sinensis* in the order Hypocreales. Both sequences of *Hirsutella* sp. isolates ARSEF8378 and ARSEF8679 were 87% identical to the ITS sequence of *Cordyceps* sp. isolate BCC28585 (561bp; GenBank Accession Number: GQ250017) and 86% identical to the ITS sequence of *O. sinensis* isolate HMAS: 173795 (574 bp; GenBank Accession Number: EU570934).

### Phylogenetic analysis

The rDNA ITS1, 5.8S, and ITS2 sequences of the isolates ARSEF8378 and ARSEF8679, those of fifteen related species of *Hirsutella*, one isolate of *Cordyceps*, one of *Ophiocordyceps*, and the outgroup *Beauveria bassiana* (Balsamo-Crivelli) Vuillemin (Ascomycota: Hypocreales) were used to compile a data matrix for the maximum parsimony analysis (Figure 2). The tree that resulted from the analysis is presented in Figure 2 and was rooted with the outgroup *B. bassiana*. The species of *Hirsutella*, together with the sequences corresponding to *Cordyceps* and *Ophiocordyceps*, were clustered in a monophyletic group, which was supported by the boostrap (99%), except for *H. longicolla* FJ973071.1. The sequences that belonged to the isolates described in this work were clustered both together and separately from the rest of the *Hirsutella* species (100% boostrap). Several species of *Hirsutella* were clustered in pairs, such as *H. gregis* and *H. kirchneri* (100%), *H. uncinata* and *O. sinensis* (95%), *H. aphidis* and *H. nodulosa* (100%), and *H. minnesotensis* and *H. thompsonii* (97%). All these small clusters were included together in a major cluster supported by a 96% bootstrap.

### Infectivity tests

*D. kuscheli* adults inoculated with conidia of isolates ARSEF 8378 and ARSEF 8679 of *Hirsutella* sp. and cultured on SDAY 1% died by fungal infections. Thirteen insects inoculated with the isolate ARSEF 8378 and 15 insects inoculated with the isolate ARSEF 8679 died 10 days after inoculation. Mycelia emergence was confirmed on 6 insects (46%) that were inoculated with isolate ARSEF 8378 and on 3 insects (20%) that were inoculated with isolate ARSEF 8679. In all the cases, mycelia emerged from the insect cadavers 12– 24 hr after insect death, and synemmata were observed within three additional days. The macroscopic development of both fungal isolates on the host was similar, though some synnemata of *Hirsutella* ARSEF 8378 appeared to be ramified.

## Discussion

The morphological features observed, plus the measures of synnemata, conidiogenous cells, and conidia of the fungi isolated from mycotized planthoopers and psocids ARSEF8378 and ARSEF8679, suggest that they most probably belong to the genus *Hirsutella*. Additionally, the morphology observed is quite similar to that described by Speare by Mains (1951) and Meyer et al. (2007) for *H. citriformis*. Nowadays, morphological characteristics are not enough to define the identity of organisms; therefore, we decided to amplify and sequence a DNA fragment containing the ribosomal ITS. Such sequences proved to be highly homologous to those of species of *Hirsutella*.

Furthermore the maximum parsimony analysis resulted in a cluster showing there was a close relationship between the organisms we isolated. Furthermore, they were part of a major cluster that included many different species of *Hirsutella*. This group included quite diverse organisms, which is in accordance with previous reports (Boucias et al. 2007b; Meyer et al. 2007). All the evidence confirmed that the two fungal isolates that parasite insects are representatives of *Hirsutella*. Even though the morphological characteristics suggest that they might be *H. citriformis*, the lack of annotated ITS sequences corresponding to representatives of this species prevent us from accurately establishing the species of *Hirsutella* the isolates belong to. More DNA sequences should be analyzed to clearly define their identity.

Surprisingly, *Hirsutella huangshanensis* C.R. Li, M.Z. Fan & Z.Z. Li, the anamorph of *Cordyceps formosana* Kobayasi & Shimizu, was clustered close to both isolates, though with low boostrap values (64%), which suggests that the cluster was poorly supported. This is not surprising considering that their morphological characteristics are quite diverse.

There are various reports about new species of *Hirsutella* with morphological characterizations, but few reports have verified the infectivity of this species against insect pests. *H. citriformis* was reported as a pathogen of *Diaphorina citri* Kuwayama by Meyer et al. (2007) using field-collected infected insects and fungal cultures on rice as source of infective conidia. They reported that all insects died by 6–9 (-10) days post-inoculation. On the other hand, Casique Valdéz et al. (2011) also evaluated the pathogenicity of *H. citriformis* against *D. citri* and *Bactericera cockerelli*. While testing the ability of *Hirsutella* isolates to infect insects, we found that the time elapsed from the moment of inoculation until insects died was similar to that described by Meyer et al. (2007).

This is the first report of *Hirsutella* sp. as a pathogen of *D. kuscheli*. These preliminary results are promising considering the development of biological tools because both isolates of *Hirsutella* proved to parasite insects *in vitro*. Although more experiments remain to be done, the isolates have the biotechnological potential to be tools to control insects such as planthoppers and psocids, though additional studies aimed at establishing the spectrum of organisms they can control should be done. Whereas this fungal species is fastidious and requires specific condirions to develop *in vitro* cultures, studies are needed to investigate additional culturing methods with the aim to increase the speed of mycelial growth and create abundant sporulation of the pathogen in order to develop an appropriated formulation to control insect pests.

##

**Abbreviations**
ITS: internal transcribed spacer
